# A Pathological Inquiry into the Nature of Hydrocephalus, Grounded on Symptoms and Morbid Anatomy

**Published:** 1829-01-01

**Authors:** 


### A PATHOLOGICAL INQUIRY INTO THE NATURE OF HYDROCEPHALUS. &c. By *Thomas Mills*, M.D. &c.

[Dublin Hospital Reports.]

IN forming rules for medical practice two plans have been adopted, which are widely different. By some they have been deduced from experience, while with others experience has been founded upon them. The patience and research, which are necessary to trace effects to their legitimate causes, and to collate such a variety of particular facts as authorise general conclusions, are seldom met with ; and, as it is much more easy to construct a theory and then form a rule, than to study Nature in all her intricacies, and draw from solitary and distant observations, inferences which may guide our future conduct, we are doomed to wade over ten volumes of undigested, unmeaning verbiage, for one out of which we can extract improvement, and upon which we can safely depend.

Our present author is a steady and successful student of the Baconian philosophy, and, although his paper upon hydrocephalus presents little either in theory or practice which was not previously known ; yet by testing with nature, our knowledge of a very obscure disease, and by exclusively advocating what experience has confirmed, Dr. Mills has conferred an important favour on his profession. He, who had hitherto rested satisfied with theory, and had assumed as granted what he had not the industry to prove, will now have the *probanda* furnished to his hand ; and he, who had adopted doctrines insufficiently supported, will have, in the present and former communications of this gentleman, on diseases of the brain, more than the authority of a name, or the sophistry of a theory, for changing his opinions.

The first and greatest part of this Essay is devoted to the history of cases, with comments upon their treatment and results ; and the pages which are appropriated to general remarks, are given as the fruit of the previous histories. We shall not, however, review *seriatim* each individual case, as many of them are similar ; but, beginning with his general observations, insert, as we proceed, by way of illustration, such as are most interesting, either for the peculiarity of their symptoms, or the importance of their treatment. Of the twenty cases detailed, twelve were of the acute form, and ran their course to a fatal termination in a very short period ; six are adduced as illustrations of the chronic variety, and the last two are given as supposed instances of recovery.

After making a few very just observations on the value of dissection, as a source of pathological and therapeutical knowledge, our author enters upon his subject by stating his objections to the term hydrocephalus, as expressive of those symptoms designated by that epithet.

"The name of hydrocephalus seems an improper one, inasmuch as it leads to the employment of remedies calculated only to excite the action of the absorbent vessels, and removes from view the nature of the complaint in that stage in which perhaps it is alone curable. We thus take the name of the disorder from the consequence of arterial excitement or venous congestion, viz. watery effusion, instead of deriving it from the inflammatory action itself. Might not the acute species arising from inflammatory action be properly denominated hydrocephalitis ?" 353.

There is no denying the existence of that association of ideas which attaches to all forms and varieties of dropsy, weakness of fibre, and want of strength, and the repeated efforts, which have been made by successive writers on this affection, to select a more appropriate title, prove, independent of every argument which can be drawn from more important sources, how unsatisfactory the present is considered. M'Bride calls it *hydrocephalic fever ;* Cullen, *apoplexia hydrocephalica ;* Porter, *encephalitis ;* Rush, *phrenicula* and *chronic apoplexy ;* Quin, *dropsy of the head ;* Smyth, *hydrencephalus ;* Good, *cephalitis*

*profunda*, and *hydrops capitis;* and many others "*water in the head.*" But, without stopping to discuss the merits of each proposal, we may observe that, long before the appearance of Dr. Mills' paper, we had chosen for our own private nomenclature the very term, (with a trifling alteration) which he here proposes for adoption. *Hydrencephalitis* seems to us even less objectionable than *hydrocephalitis*, for the same reasons which induced Dr. Smyth to prefer *hydrencephalus* to *hydrocephalus;* but, as the alteration involves nothing of practical importance, we shall feel happy in seeing the present misnomer exploded, and *hydrocephalitis* employed to designate a disease which has little more analogy with dropsy than hydrothorax has with the attack of pleurisy which gave it birth. Not only the nature but the seat of this cerebral derangement is expressed by the last part of this appellation, and the first denotes the natural issue of the preceding excitement, if not checked by an active and timely treatment. The only objections that occur to us against the use of this epithet are,—that what comes last in the progress of the disease, stands first in the formation of the name; and that the existence of serous effusion is neither universal in its occurrence, nor essential to the production of those symptoms which have generally been regarded characteristic. These objections, however, are mere *niceties;* and believing, as we do, in opposition to Monro, and every other writer of the same school, that acute hydrocephalus ought to rank with the phlegmasiæ, the circumstance of this term having the same ordinal termination with the diseases therein arranged, we consider of primary importance; and, although the sensorial structure may be so disorganized before the stage of effusion shall have arrived, as to produce a cessation of all vital function, yet the natural termination seems to be effusion, and every instance in which it does not occur, we consider merely illustrative of the fact, that the phlogistic excitement, in such cases, has been so active and so rapid as to destroy life before the arrival of the last stage.

"Hydrocephalus presents itself under two forms; the acute and chronic. In the acute, which is the more frequent, it commonly lasts from seven to twenty-eight days; in the chronic, from one to six months. This is the usual duration of the complaint, as far as my experience goes; but as in the latter form it may occasionally be protracted to one, two, three, or even sixteen years, so, in the former it may run its course in three or five days." 434.

The acute variety has been usually divided into three stages, but Dr. Mills confines himself to two; and, as his arrangement is more simple, and perhaps as useful, we cannot err far in permitting this division.

In the first stage appear languor, lassitude, dullness and pain, vertigo, or heaviness of head. The eyes are inexpressive, or intolerant of light; the ideas are confused;—the temper becomes fretful; there is a disposition to retirement, and a disinclination from business;—the stomach is irritable, occasioning nausea, retching, and vomiting;—the tongue is generally moist, but sometimes dry;—the skin is dry and hot, sometimes sallow and cool;—the urine is light coloured, especially when the skin is dry;—when perspiring, it is red and often turbid;—the bowels are constipated, and the pulse varies in strength and frequency, being sometimes strong and quick, at others, weak and slow.

In the second stage of disease the balance of the mind is lost, languor and lassitude yielding to stupor and delirium;—the head-symptoms become more marked and severe;—the pain is often excruciating, causing the patient to start and scream;—the eyes are dull and cloudy, often dim and blind;—the pupils are contracted, or dilated, sometimes alternately;—strabismus is more apparent than during excitement;—picking of the nose is a common symptom, and the fingers are frequently thrust up the nostrils with violence;—the complexion of the face changes much, being now red and then pale;—the lips and eyes are often surrounded by a blue circle;—hearing and smelling are blunted, as well as the sense of sight;—the voice, which was at first commonly hoarse, becomes shrill, and towards the close low and indistinct;—the head is often thrown back and rendered stiff by spasm of the muscles of the neck;—the respiration, which was hurried in the first stage, becomes laboured, and the patient sighs deeply;—the stomach is very much disturbed, yet the appetite is very variable, being sometimes voracious and sometimes lost;—the bowels are very costive, and the fæces vary from a yellow curdy mass, to the colour of chopped spinage;—the skin is usually cool and damp, and sometimes so irritable as to suffer much pain when touched, at other times it is quite senseless;—the muscles are variously contorted and convulsed, the hands tossing about the head, the eye-brows corrugated as though with pain, and the features distorted into a multiplicity of aspects;—the excretions are passed involuntarily, and life, at length exhausted by the struggle, vanishes in universal convulsions. P. 435—41.

While the phenomena now enumerated are generally met with in hydrocephalus, some of the most distinctive are occasionally absent, and some are frequently modified. Thus, head-ache is sometimes wanting, and sometimes it is restricted to one hemisphere. The first of the following cases is an example of the one peculiarity, and the second furnishes an illustration of the other.

*Case* 4. May 28th, a girl, æt. 3, after some previous indisposition, began to complain of heaviness of head, drowsiness, loss of appetite, sighing and languor. Face flushed, fæces green, and abdomen uneasy under pressure; *calomel and scammony, purging enema, and saline mixture.* June 4th. Had been somewhat relieved by her medicines, but to day, vomiting, thirst, and restlessness came on: pulse 126, irregular, and weak; *calomel repeated, and* 10 *leeches to temples.* 6th. Symptoms improved; *continue.* 9th. Return of fever, vomiting, restlessness, and other bad symptoms; countenance dejected, tossing of the arms; *blister to nape, and powders repeated.* 12th. Little change; *continue.* 14th. Strabismus, moaning, screaming, pupil of the left eye dilated, face pale and flushed alternately, fæces " like to chopped parsley," tossing of arms, pain on pressing abdomen, pulse 126, irregular; 10 *leeches to temples, cathartic mixture and tepid bath.* 15th. Symptoms better; *blister nape.* 16th. Convulsions of upper extremities, respiration hurried, sight of left eye much injured, pulse 132; *enem. fœtid. c. tinct. opii.* 18th. Convulsions general, sight of both eyes gone, right pupil contracted, left dilated, screaming and moaning:—died at night.

*Dissection.* Vessels of the brain much distended, some effusion between the arachnoid and pia mater, and ventricles contained ℥iij. of watery fluid. Other parts of body healthy.—*p.* 420-21. In this case there was no disease *without* the head, yet no headach was complained of during its entire course; proving that much cerebral disease may be going on, and that even death itself may occur from vascular excitement *within* the head, without producing pain, or serious uneasiness. In the second case there was severe head-ach, but it was confined to one side, which exemption is accounted for by appearance after death.

*Case* 3. December 4th, Miss P. æt. 8, complained of pain of left side of head and retching, lassitude, chilliness, restlessness, anorexia and peevishness; pulse 66, regular; 10 *leeches to temple, and a powder of calomel and scammony every* 4 *hours.* 5*th.* Leeches gave short ease, three dark stools, urine turbid, pulse 62, regular, pain still confined to left side of head, but less severe, languor, heaviness, oppression and palpitation; *powders repeated, and* 8 *leeches to the left temple.* 6*th.* Acute headach, four dark stools—face alternately pale and flushed, pulse 60: *Blister to nape, and* 12 *leeches to temples.* 7*th.* Headach less severe, frequent retching, moaning, sighing, and restlessness with much picking of the nose; pulse 66; *repeat powders and give a pill of opium and calomel at night.* 8*th.* Some rest, skin hot, pulse 72, other symptoms unchanged; *blister left temple and repeat the pill; oil and enema.* 9*th.* Pulse 80, delirium, pain in left hemicranium continues, as do the other symptoms; *pill repeated, and* 3 *grs. of James's powder every* 4*th hour.* 10*th.* Stupor and delirium; 12 *leeches to temples, blister to occiput and cathartic powder.* 11*th.* Some ease from leeches, but screaming and moaning continue, pulse 94, fæces dark, skin hot; 4 *grs. of James's powder every 3d hour.* 12*th.* Sight impaired, pulse 114, stupor and delirium; *powder repeated and blister to vertex.* 13*th.* Slight convulsions, pulse 122; *opium pill and cathartic powder resumed.* 14*th.* Pupils dilated, dejections involuntary, pulse 132, irregular and feeble; convulsions, delirium and imperfect vision continue; *opium pill at night.* 15*th.* Died convulsed.

"*Dissection.* Dura mater highly vascular, and covered with a serous fluid; minute florid arteries and turgid veins are distributed throughout this membrane. Upon the left hemisphere of the brain the veins are excessively turgid, and numerous red points are observed in its substance. Along the course of the vein which returns the blood of the middle cerebral artery, a thick purulent fluid is found deposited at different points between the pia mater and the arachnoid membrane. Cortical part of the brain of a very dark colour. Left lateral ventricle highly vascular, enlarged to nearly four times its usual size, and distended with about three ounces of a watery fluid. Right hemisphere of the brain not so vascular as the left, but the same vein as in the left hemisphere is opaque, and a similar purulent fluid is apparent, but not in so many points. Right lateral ventricle as vascular, and nearly as large as the left. Plexus choroides excessively vascular. Base of the brain loaded with numerous minute florid vessels. Optic nerves surrounded at their juncture by a serous fluid contained in the cells of the arachnoid membrane. Pons Varolii exhibits on its surface the same anasarcous appearance as that which is observed round the optic nerve. About two ounces of a watery fluid are found at the base of the brain, and in the theca vertebralis." 111.

How will Dr. Monro account, on his principle, for the presence of pus in this case between the membranes? It is not a dropsical deposit! and no one will question the identity of this history with that of the purest case of hydrocephalus. We could have wished that Dr. Mills had examined whether the serum found in the ventricles would coagulate by heat.

"This disease often attacks the healthy children of healthy parents, but occurs more frequently in the puny or scrofulous, or in children whose parents are scrofulous, debilitated, or worn out by intemperance. There were appearances of scrofula in twenty-two of the patients examined; in two the brain appeared scrofulous; in three the lungs; the liver in four; the mesenteric glands in eight; the spleen in four; and in five the cervical glands. Of the patients who recovered six had no visible marks of scrofula, and the parents of twenty-six were in appearance free from any affection of this kind." 435.

Percival states that out of 22 cases, 11 were decidedly, and four apparently scrofulous; and Cheyne records two instances where five out of one family, and 10 from another fell victims to this disease. Such melancholy occurrences have given birth to the suspicion that hydrocephalus is a scrofulous disease, and always hereditary. But we are unacquainted with any fact, either in its symptoms, treatment, post-mortem appearances, or habitudes, in support of this view. The frequent coincidence of hydrocephalus with a strumous habit establishes, we believe, the existence of a predisposition in such systems to such a disease; but that hydrocephalic action is essentially scrofulous, is neither supported by its symptoms during life, nor by the structural changes which dissection develops after death.

It has, also, been very fashionable, of late, to regard hydrocephalus as arising from, and depending upon, a deranged condition of the chylopoietic organs. Before the days of Cheyne, chylopoietic derangement was seldom even mentioned in the general catalogue of exciting causes; but since his Essays have appeared, in which he called our attention to a source of evil that had been almost overlooked, every succeeding writer has insisted upon its importance, and some have even gone so far as to assert that few cases will be found which cannot be traced to this source, if minutely examined.

Now, we mean not to derogate from the important sympathies of the chylopoietic organs, when we refuse to ascribe to them such extended influence. We believe that a disordered state of the stomach, liver, and intestines, will derange the health in general, and the cerebral functions in particular, and that a frequent repetition, or long continuance of this derangement will favor, and may occasionally induce structural disease within the head; but we believe not that such an effect is of so frequent occurrence, nor that a majority of cases is to be ascribed to this cause. If the instances, which have been supposed to arise from it, had been attentively watched from the commencement, we hesitate not to say that the opening symptoms of most of them would have been found to belong to the head, and that the abdominal disorder was merely the effect of previous disease. In children, who are mostly the subjects of this affection, and who cannot describe with accuracy either the introductory signs of indisposition, or the seat of the first feeling of uneasiness, symptoms of disordered bowels are much more palpable to the spectator than those of a disordered head; and while the practitioner may be unable to discover the existence of headach, he has little trouble in recognising, by nausea and vomiting, a deranged stomach. This is a subject of importance and should, therefore, be seriously considered, and we are glad to find that Dr. Mills has not permitted it to escape unnoticed.

"I have been led to conclude from a view of the cases above stated, and the appearances presented on dissection, that in Hydrocephalus the brain is the organ primarily engaged; but because nausea, retching, diminution of appetite, foulness of the bowels, a morbid state of the secretions, and pain or uneasiness in the hypochondria, are usually observed in the first stage, some have taken a totally different view of the disorder, and have conceived that it originates, not in a disease of the brain, but in a derangement of the chylopoietic viscera;"

And, after making some remarks unfavourable to this view, he very justly adds—

"We may easily conceive how fatal the consequence must be of mistaking such a complaint as Hydrocephalus; for while directing our attention to the removal of secondary symptoms, the affection of the brain advances, and if effusion take place, becomes for the most part incurable. The time thus lost is for ever to be deplored. Sometimes, indeed, temporary relief is obtained by the use of alteratives and cathartics, and the practitioner indulges a hope that he has performed a cure; but a little time and the delusion vanishes. The deep sigh, the moan, the scream, the fixed stare and the convulsions, announce Hydrocephalus in all its terrors, and so far advanced, as to baffle the skill of the most experienced. In truth Hydrocephalus is a disorder so insidious and dangerous, as to demand at once the acumen and decision of the practitioner; not a day, not an hour should be lost before we have recourse to the prompt and judicious employment of our most efficient remedies. Even in cases somewhat equivocal we may with reason ask,—Is the physician justifiable in neglecting to have recourse to measures calculated to obviate effusion, more especially when we know that these measures cannot materially injure the patient." 445.

Hydrocephalus frequently appears without any known cause, and without exciting a suspicion of its approach by any premonitory symptoms; but it may arise from injuries inflicted on the head during labour, or after birth, from malformation and abscesses in the brain, from exposure to cold and wet, from sudden vicissitudes of climate and insolation, from obstruction in the thoracic, or abdominal viscera, from a strumous habit, or hereditary taint. In short, whatever will unhinge the balance of the circulation, increase the quantity of blood in the head, or stimulate its vessels to inordinate action, may operate in producing this disease. It is a curious circumstance, that mental agitation in the mother during pregnancy often imparts to her child a predisposition to hydrocephalus; "In the year 1809," says Golis, "when our imperial city (Vienna) was bombarded, most of the chidren, who were born after this frightful catastrophe, in about 10, 20, or 30 days after their birth, were seized with convulsions and died. Within the cranium were found traces of inflammation, and in the ventricles of the brain, effusion of lymph and serum;" and it is a well known fact that children, whose mothers had been distressed during their pregnancy with symptoms of cerebral congestion, have been much more frequently than others attacked with hydrocephalus, shortly after birth. But these observations are not merely curious; they are important. They demonstrate the intimate connexion which exists between the maternal and fœtal systems, the influence which mind exerts over matter, and the tendency to an hereditary character of every cerebral disease. They prove of what importance it is to be intimately acquainted with the parents' mental and corporeal predispositions, as well for the prevention as removal of infantile disorder, and how careful we ought to be in noting each morbid phenomenon at its earliest appearance, in those who have been thus born under suspicious circumstances.

There exists in infancy a lively susceptibility to disease. Every organ at that youthful age is as sensitive to impressions as it is unable to resist them, and the head especially, is most obnoxious to disorder, from the delicacy of its structure, and the large proportion of fluids which it circulates. The following interesting case will forcibly illustrate the truth of this observation—

*Case 5.* A boy, aged 7, was attacked on the 21st of May, with the ordinary signs of hydrocephalus, and, after having improved under local and general bleeding, purging, blistering, and calomel, dyspnœa, palpitation, anasarca, ascites, and pain in the right hypochondrium, supervened about the 10th of August, and he died on the 23d. As the dissection constitutes the interest of the case, we will give it at length in the words of the author.

"General effusion of a serous fluid between the arachnoid membrane and pia mater. Surface of the brain highly vascular. About two ounces of a serous fluid are found in the ventricles and at the base of the brain. Considerable quantity of serous effusion in the arachnoid membrane, around the junction of the optic nerves.

"Abdomen.—Recent and old adhesions between both lobes of the liver and the diaphragm, and the parietes of the abdomen. Three pints of a serous fluid in the cavity of the abdomen. Both lobes of the liver enlarged, hardened, and more vascular than natural; externally their surface is dotted with numerous small, whitish tubercles; internally are several small tubercles containing a cheesy matter. Peritonæum and omentum dotted with small white tubercles of the size of grains of sand. Spleen highly vascular, adherent to the diaphragm, and internally and externally studded with tubercles.

"Thorax.—Right lung almost converted into brownish-white tubercles, and adherent throughout to the parietes of the thorax. Left lung partly adherent to the mediastinum, pericardium, and pleura costalis; the surface and internal portion of this lung are studded with small cream-coloured tubercles. Pericardium considerably thickened, and coated internally with a layer of coagulable lymph; this layer is extended over the surface of the heart, destroying its natural, and giving it a honeycomb appearance. The pericardium contains about an ounce of serous fluid. Heart enlarged to twice its usual size. Right auricle thickened. Right ventricle larger than natural. Left auricle diminished in size, and thickened. Left ventricle thickened and contracted." 421.

It is easy to detect the presence of hydrocephalus when it has arrived at its last stage. The heavy sigh, deep moan, screaming, tossing of the hands around the head, derangement or loss of the external senses, state of the pupils, position of the head, convulsed or palsied state of the muscles, frequent vomiting, peculiar aspect of countenance and color of the stools, are too unequivocal, especially when most of them are present in the same case, to deceive. It is at the beginning, or during the period of excitement, that any difficulty will occur; but as Dr. Mills very properly observes—

"The presence of Hydrocephalus cannot be judged of from one or two symptoms, but from a general survey and minute examination of all the symptoms and circumstances of the case. We should be acquainted with the constitution and habits of the parents of the patient, his temperament and constitution, his previous diseases and their effects, and the past and present state of the secreting organs, more particularly of the skin. This knowledge will aid the practitioner in forming his opinion; but the symptoms and circumstances which appear to me to characterize the disease in its first stage are · a peculiar expression of countenance, indicative of oppression, pain and despondency; frequent sighing; a disposition to retirement; a heat, weight, pain or heaviness of the head, or all these combined; intolerance of light; waywardness and fretfulness; a low irregular fever; frequent nausea or retching; an irregular state of the appetite and bowels; and the continuance of the disease, notwithstanding the employment of aperients and the remedies usually prescribed in the disorders of children." 447.

The state of the pulse has been considered, by a great majority of practitioners, as the surest guide we have in indicating to us the several stages of this disease. In the first, we are told, it is quick and strong; in the second, slow and full, and in the third, rapid and irregular. But we are convinced there is much error in this doctrine, and that a belief of it has led to the very worst consequences. Quin has observed instances in which there was neither vomiting, aversion to light, "nor change of pulse;" and we have seen many cases in which the character of the pulse never varied, at least, to an extent which would entitle the above arrangement. As the *sixth* case in the present Essay will, however, confirm this assertion, we will not leave our author in quest of proof, and, having another object to serve in adducing it, to which we will subsequently allude, we will not confine ourselves to a mere history of the pulse, but give a digested view of all the symptoms and appearances on dissection.

*Case* 6. Dec. 6th, Miss M. æt. 7; a very healthy child, was suddenly seized with shivering, headach, nausea, vomiting, and pain of right hypochondre; pulse 116, of good strength, skin hot and dry, tongue foul, no appetite, and thirst. *Bled to* ℥viij. *calomel and jalap.* *7th.* Headach slightly relieved, pain, especially in occiput, much vomiting, face flushed, three green stools; pulse 120, strong; *bled to* ℥vj. *blister nape, powder repeated.* *8th.* No relief; pulse 112, 24 *leeches to temples, powder and mixture for bowels.* *9th.* No relief from leeches, great weight of head and delirium—face alternately pale and red, pulse 124; five green stools, aversion from moving head, lies chiefly on back, loss of taste and smell; *bled to* ℥viij. *pill of opium and calomel, cathartic powder, blister occiput.* *10th.* Pulse 114; symptoms the same; 12 *leeches to temples, pill and powder repeated.* *11th.* Pulse 124, much retching, moaning, stupor and restlessness—face and trunk hot, while lower extremities are cold; inability to raise head; five stools, green; pupils natural, but vision hurt; *calomel and Dover's powder.* *12th.* Screaming "oh! my head," delirium, stupor; pulse 120, feeble and irregular; *blister head—powder repeated.* *13th.* Breath mercurial—urine copious—several stools—retching and deafness continue; 20 *leeches to temples, powder renewed.* *15th.* Ptyalism, jaws swoln, headach; pulse 110; pupils natural, and sight restored; *infusion of senna, friction with mercurial ointment, and powder to be repeated without the calomel.* *17th.* Screaming, moaning, and delirium; unwillingness to move head; face alternately pale and flushed; pulse 120, feeble and irregular; *medicines repeated.* *18th.* Eyelids weak, pupils contracted, vision good, bowels open; (does ptyalism continue?) *Repetr.* *19th.* Died this morning.

*Dissection.* Dura mater vascular, serum under arachnoid, surface and substance of brain too vascular, lateral ventricles contained ℥ijss. of serum, plexus choroides turgid, and walls of ventricles very vascular, cerebellum too vascular, pluræ of both sides adherent, no particular disease of any other organ.

Now, since there can be no doubt as to the real hydrocephalic nature of this case, as well from the symptoms by which it was attended as from the post-mortem results, and since the character of Dr. Mills is above suspicion, either for misrepresentation or misstatement, there can be no denying the *possibility* of the occurrence—that the pulse may not fall during the entire course of this disease. And, having established such a possibility, we would recommend practitioners to be more guarded in holding this arterial vaccillation as pathognomonic of effusion, and as decisive of death.

The value of an appropriate and expressive epithet is more easily estimated in the treatment than in the nosology of a disease. Mere nomenclature, when valued on its own account, is more a subject of taste than of talent, and respects more rigidly the exactness of science than the exigencies of practice. But in medicine, names ought not to be arbitrary. In taking an etymological view of hydrocephalus as a disease, we look more to its result than to its essence;—more to the proceeds than to the nature of morbid action. We consider it as "water in the head," without reflecting on the manner of its getting there, or on the connexion which it may have had with preceding states and stages of disease. We confine our attention to a mere *accident*, and, overlooking the sum and substance of the disorder, adapt our remedies to what need not, and to what does not, frequently exist. We repeat it, and we wish to impress it upon the attention of our readers, that the disease which we call hydrocephalus, is not necessarily dropsical, nor essentially a dropsy. It differs from the order of hydropes as much as pleurisy does, and it were just as philosophical nosology to rank inflammation of the thoracic lining with water in the chest, and just as judicious therapeutics, to subject them to the same treatment, as to enrol hydrocephalus with hydrothorax, and doom it to a similar plan of management. Atony and weakness are not the real characters of this disease, nor will it be cured by tonics and diuretics; and when symptoms of effusion appear, they appear only as the successors of a more acute stage, and as the consequences of strength that has been overpowered in the struggle, and has had recourse to effusion as the last attempt to get quit of an undue excitement. As a general direction, therefore, would we say, depend on nothing but what will *prevent* effusion; diuretics and tonics can only *remove* it; and wait not till symptoms of effusion appear, for effusion may never come, and yet life perish; but, with the first manifestations of disorder, have recourse to such remedies as blood-letting, cathartics, antimonials, mercurials, and blisters. We must look to these as our principal, if not sole hope, and be neither paralyzed with the horror of effusion on the one hand, nor, upon the other, be deceived with the imagination that a few purgatives and tonics, by improving the stomach and bowels, will always, or often remove such an affection as hydrocephalus.

"In a disease of so dangerous a tendency it is scarcely necessary to state that the loss of a day, perhaps of an hour, may be followed by fatal consequences. The temporal artery, the jugular vein, or a vein in the arm, should be opened, and the blood allowed to flow until some impression is made on the general habit, and until the morbid actions of the vascular system of the brain are modified, or totally changed. That such an effect has taken place may be known by a paleness of the countenance, a shrinking of the features, and a tendency to deliquium; or by a diminution or removal of the heat, pain, weight, or uneasiness of the head. As soon as this is produced, our object, for the time is accomplished, and the orifice should be closed. Immediately after, or even previous to venesection, brisk cathartics should be administered and repeated at short intervals; and these I recommend not merely from their emptying the bowels of their contents, but because they act on the exhalants of the entire alimentary canal. Thus we lower the tone of the heart and cerebral vessels, and promote a more equal distribution of the blood." 448.

As to blood-letting, both the quantity taken, and the mode of taking it, must depend on circumstances;—in very young subjects, leeches will be most applicable; but if the patient be of an age at which the lancet can be used, general depletion will be highly serviceable in conjunction with leeches, or cupping. Dr. Mills recommends each bleeding to be carried nearly to deliquium, in order that some permanent impression may be made upon the circulation in the brain; and with this advice we entirely concur. We have found that, during the first stage, while the vascular system is strongly excited, children will endure the removal of a much larger quantity of blood without fainting than could at first view be conceived; and, therefore, if we measure our depletions by rules constructed upon principles of health, upon the age of the patient, or the delicacy of its system, we will only be sinking the strength without subduing the disease. Leeches may be applied to the temples, vertex, or behind the ears, or in more advanced patients, cupping-glasses may be beneficially substituted; and when the lancet has been employed, it will be found good practice to apply a few leeches to the head, immediately upon tying up the arm. The general bleeding prepares the way for a topical abstraction, and the topical abstraction confirms the effect of the general bleeding.

Brisk cathartics are to be administered regularly, especially at the outset, and during the first period of disease; for the quantity of feculent matter contained within the intestines, in many cases, is really surprising. One instance now occurs to us. The patient was a young lady, who, after an attack of fever, during which head-symptoms predominated and had not been opportunely nor sufficiently overcome, was seized with all the signs of water in the head; and as her bowels had been rather disposed to astringency throughout the fever, they became exceedingly torpid, indeed, almost unmanageable, on the establishment of hydrocephalus. Five and six common doses of drastic purgative were required before the intestines would answer, and figured and bulky stools were daily passed for three weeks under this stimulation, without any solid food having been taken during that time.

"As soon as a check has thus been given to the disorder of the head by the action of these several remedies, some benefit is to be expected from the judicious administration of calomel with opium. The good effects of a combination of these remedies seem to depend on their power of equalizing the circulation, increasing the secretions, and exciting the actions of the cutaneous vessels, in consequence of which the congestion of blood in the brain, or in any other organ, is diminished or removed. The dose must be regulated by its effect, the constitution of the patient, and the violence of the attack Although, generally speaking, it is difficult to induce ptyalism in this complaint, yet, as calomel in some cases unexpectedly attacks the salivary glands, and produces ulceration and mortification of the fauces, I prefer giving it at first in small quantity, which may be increased at pleasure. In acting thus cautiously we avoid the painful consequences sometimes observed to follow its exhibition.

"The efficacy of opium, united with calomel, depends, in some measure, on its checking the tendency of the latter to run off by the bowels or salivary glands; so combined, it often procures tranquil rest, determines to the surface, and allays that painful irritation of the nervous system and of the mind and body, which so uniformly accompanies this disease. I cannot bring to my recollection a single instance in which this remedy, when judiciously exhibited after depletion, was followed by disagreeable consequences; and in cases unaccompanied by great irritability of the stomach, its powers are occasionally increased by the addition of small quantities of ipecacuanha, or antimonial powder." 451.

The difficulty of producing salivation is a great peculiarity in this disease. The absorbent system seems to suffer earlier than any other, and much more than its proportion of the mischief resulting from hydrocephalic action; for, although the torpor of the intestines, the dulness of the senses, and the stupor of the mind, indicate much nervous derangement, all these phenomena generally appear later in the chain of consequences, and are in proportion to the insensibility of the absorbents. Hence it is, that the *vitality* of this system constitutes one guide by which we are aided in prognosticating the issue of the cases we attend, seeing that if we can affect them, or, in other words, produce salivation, we are sure of relieving the symptoms, and, in general, of removing the disease. The sixth case of Dr. Mills, which we have already detailed, forms an exception, however, to the last part of this remark; for, although a decided improvement followed the appearance of salivation, the patient ultimately died. But in this instance the torpor of the general system was not so extreme as in the majority of cases; the bowels yielded readily to the action of purgatives, the pupils were not at any period dilated, and there was such acute sensibility of the skin, that the application of the finger to any part of it, gave considerable uneasiness. This is the only case, so far as recollection serves us, on the progress of which ptyalism exerted no influence; for although there was a slight alleviation of the symptoms on the day preceding, and on that of its occurrence, the patient struggled only three days after it appeared. Dr. Mills remarks, that ptyalism is "so unusual an occurrence in hydrocephalus, that of the many cases I have met with, this was the only one in which mercury produced this effect." 377.

"Among the auxiliary remedies the tepid bath holds the first place: its use is often attended with manifest advantage; indeed, in some instances the benefit resulting from its employment is as unexpected as it is extraordinary; I have seen it at one time reduce the intense heat and dryness of the skin, at another, produce a genial warmth and moisture of the surface, and thus allay irritation, diminish the action of the heart and arteries, and check, for a while, the violence of the disorder. The tepid bath will prove most serviceable after the employment of blood-letting and cathartics; it should be used twice or thrice daily, according to the urgency of the symptoms, and the effect produced. The period of immersion is from fifteen to forty minutes, about an hour before which, the patient should take a dose of calomel and opium, and while in the bath, small repeated draughts of tepid whey or barley-water, in which is dissolved tartrate of antimony,* in doses so minute as not to excite nausea or vomiting; and during this period friction must be constantly applied to the body and extremities with the hand or a soft brush. Blisters are never to be employed previously to depletion, or while there is a high degree of excitement, for under such circumstances, by their stimulating powers, they rather tend to aggravate than diminish the disease. On some occasions they are useful when applied to the head or its vicinity, chiefly on the principle of counter-irritation, and partly by the fluid abstracted from the cutaneous vessels, whereby the congestion of the interior is in some measure diminished. Blisters sometimes afford relief when applied between the shoulders, or along the spine, owing, in a great degree, to the connexion of the spinal marrow with the brain, and instances occasionally occur where evident advantage proceeds from their application to the extremeties. Now, as in these cases I have observed perspiration to follow their employment, the benefit hence resulting seems to me to proceed from their action on the vessels of the surface, and the power they thus possess of restoring the equilibrium of the circulation." 454.

Our author strongly recommends the external and internal employment of tartar emetic. Externally, as an ointment, he has found it powerfully useful in such cases as arose from repelled eruptions. By recalling, as it were, the old disease in a new and more manageable form, the balance of the circulation is restored, and the head is relieved of its excessive quantity of blood. Internally, he advises it in the same doses and with the same views as our Italian neighbours; and, from the control we have seen it exert in phrenitis, mania, and other analogous diseases, we have no doubt but nauseating doses repeatedly administered, so as to keep up the effect for some time, will, by determining to the surface, eliciting the secretions, and sinking the powers of the circulation,

* "Since this paper was written, (1821,) I have given tartar emetic more frequently, and in larger quantity than formerly, and with decided benefit, especially in the acute form of the complaint. This active agent possesses, in many instances, the power of diminishing the tone and activity of the vessels of the brain and heart, and thus of breaking the chain of diseased action. This appears from its effects: a sinking of the pulse, collapse of the features, tendency to deliquium, accompanied by cold perspiration, and a diminution of the head-ache, and throbbing of the temples. I was induced to make a trial of this remedy in hydrocephalus, from witnessing its good effects in maniacal delirium and other disorders of the brain, arising from a high degree of vascular excitement. The dose varies from one-fourth of a grain to two, four, five, or even ten grains, dissolved in water, to which may advantageously be added, when the stomach is irritable, small doses of the tincture of opium."

have a most beneficial effect. The quantity given, although small, must be greater than in ordinary kinds of inflammation, for it is a strange, but unquestionable fact, that the stomach, although most disordered and inclined to vomiting, is remarkably insensible in many cases to the stimulus of medicine,—so much so, that some have regarded this insensibility as characteristic of hydrocephalus.

There is one external remedy which the Doctor overlooks, that appears to us deserving of very particular notice; that is, the application of cold to the head. Dr. Abercrombie speaks highly of it in common inflammation of the brain, as well as in hydrocephalus, and we believe that, if used at the proper time, and to the proper extent, we are not possessed of a remedy endowed with more power, or productive of more good. It lowers excitement by abstracting heat, gives a tone to the internal vessels through their connexion and sympathy with those that are without, reduces the force of the circulation, calms the irritability of the nervous system, and disposes the mind to tranquillity and sleep.

Cloths occasionally moistened with cold water will not produce these effects, nor will a solitary shower-bath: the water is not only to be applied, but applied *forcibly.* A stream, regulated as to size by the age of the patient and the symptoms of the case, should be allowed to fall upon that part of the head most afflicted with pain, and continued until the temperature of the scalp fall to the natural standard, feelings of weakness be expressed, and relief procured. By this simple application after, of course, due depletion, we have witnessed results that no hopes could have anticipated, and from a conviction of its applicability in most instances, and of its efficacy in many, would we warmly recommend it to the serious consideration of our readers.

Too often, however, nature proves stronger than art, and medicine is baffled in her attempts to cure. The following case is worth insertion, as strongly illustrating the impotency of all means,—even the most judicious and active,—when an organ of such sensibility to excitement, and of such importance to life, becomes deeply involved in disease.

*Case* 5. Nov. 23d, a boy, æt. 12, was, in consequence of a fright, seized with head-ache, chilliness, nausea, and vomiting, prostration of strength, alternate paleness and flushing of face, hot skin, furred tongue, quick and strong pulse, drowsiness and low delirium. *Bled to* ℥viij. *powder of calomel and jalap.* 25th. Relief from bleeding, blood slightly *buffed,* and with a small proportion of serum, four stools, green and yellow, urine turbid, pain of forehead and weight in occiput, pulse 106, sighing and moaning continue; 24 *leeches to temples, scammony and calomel powder. Evening;* nausea and vomiting, frequent sighing, low delirium, unable to raise head, pulse 108, skin cool, tongue clean, speech indistinct, pupils natural, stupor so great as to prevent him from making any complaint. *Bled to* ℥xvj., *blister nape.* v. *grs. of calomel every* 3*d hour, and a purging enema every* 4*th hour.* 26th. Better, sense of weight but no pain of head, stupor, slept some, pains in left thigh and foot, pulse 102, regular and strong, vomiting, bowels free; 36 *leeches to temples, calomel and jalap.* 27th. In addition to leeches, was bled from temporal artery to weakness; stupor, delirium, and vomiting continue. Pulse 108, soft and regular, thirst, four yellow stools; 30 *leeches to temples, powder repeated, blister between shoulders.* 28th. Some sleep and more sensible, head easy, sight and hearing good, pulse 108, regular, skin cool, tongue clean, urine copious, two dark stools. *Pill of opium and calomel.* 29th. Much moaning, stupor, delirium, and double sighing, pulse 118, strong and vibrating, skin hot and dry, head reclined, left eye half closed; *blister head, pill repeated.* 30th. Screaming, grasps head with both hands, strabismus, stupor, delirium, pupils contracted, pulse 124; *pill repeated, and calomel and squill powder.* Dec. 1st. Symptoms unchanged, pulse 120; *pill renewed*, v. *grs. of calomel every 4th hour, and* ʒj. *of mercurial ointment to be rubbed in night and morning.* 2d. Pulse 126, regular, other symptoms unchanged. *Cont.* 3d. Dead.

*Dissection.* ℥iij. of serum in lateral ventricles, and ℥ij. at the base of the brain, membranes and substance of brain natural; bladder very full of urine; left lung adherent; and pericardium contained ʒij. of serum. P. 372.

The Doctor, with very good reason, reposes little faith in the operation of issues. Their effects are too tedious to be of much service in acute cases. They are better adapted for the chronic form, on the nature and treatment of which he makes a few sensible observations, to which we will now proceed.

Speaking of the symptoms peculiar to this variety he says:—

"Vertigo, confusion of ideas, heaviness, or a dull pain in the head, and stupor, attended by a pallid countenance and shrunk features, usually usher in this form of the complaint; whereas, in the first stage of the acute hydrocephalus, we find lancinating pains of the head, throbbing of the temples, suffusion of the eyes, or intense heat of the forehead. In chronic hydrocephalus the intellect is more clouded, and the expression of countenance is generally bewildered, fatuous, or like that of a person half intoxicated. In the acute, on the contrary, the countenance is often expressive of quickness and intelligence, the voluntary powers are more enfeebled, and the vital functions more oppressed. We may likewise notice a considerable difference in the temperature of different parts of the body; the forehead and extremities being, in the chronic, often cold or damp, while the trunk is little, if at all, above the natural standard; whereas in the acute the heat is more intense, and more equally diffused. Further, we find on dissection, the turgescence of the veins and sinuses of the brain much greater, and the effusion of a watery fluid between the arachnoid membrane and pia mater in larger quantity in the chronic than in the acute. The chronic, moreover, is oftener found accompanied by disorganization of the substance of the brain or its membranes." 456.

Our treatment of this form differs very little from that of the acute variety. The same medicines are necessary to both, but since disease in the one is much more active in its degree, and more rapid in its progress than the other, more time is allowed for treatment, and less active measures will in general be required. In this variety blisters will be more beneficial than bleeding; for as chronic hydrocephalus consists rather in turgescence of the veins than activity of the arteries, whatever diminishes the serum of the blood will be as productive of relief as that which removes the red globules. With this view, issues on the head and setons in the neck are extremely useful, and may, if the disease be not entirely confirmed, cut short the progress of morbid action, and will often, if confirmed, prevent it from proceeding with its natural rapidity.

It has been often disputed whether serum once effused within the head could be absorbed, or, to use more accurate language, whether any cases of hydrocephalus have recovered after symptoms indicative of effusion had appeared? But we are not aware that the agitators of such a question have any arguments stronger than mere presumption in its support. That absorbents have not been seen in the brain by many is no objection, for negatives, however multiplied, can establish nothing, and the attentive pathologist is in possession of many phenomena which are totally inexplicable, if their existence be denied. To believe that all is lost so soon as a dilated pupil, stupor, delirium, strabismus, blindness, loss of smell and hearing, supervene, is to paralyze the energies of medicine, and to throw the most insurmountable of all obstacles in the way of its improvement. It cannot be denied, that when such symptoms come on, our prospects of recovery are much contracted and obscured; but still hope has not deserted him, who, seeing no fatality in such cases, believes that cures may be effected at the *eleventh hour*. As Dr. Mills has recorded two such cases we will present them at full length, that, by having both the symptoms and treatment before him, the reader may be enabled to decide upon their identity with hydrocephalus, and upon the value of the remedies by which the patients were preserved.

"*Case* 1st. May 10, 1813. Miss C., Mary-street, æt. 12, of a delicate frame and quick perception, subject to a discharge from the ears, which has ceased for some weeks. For the last fortnight has been complaining of nausea, loss of appetite, headach, prostration of strength and restlessness. Cathartics have been administered without producing any good effect. A younger brother died about a month ago of Hydrocephalus. Pulse 124 and tense; skin hot and dry; thirst; shooting pains in the temples and forehead; pupils dilated, but they contract on the application of light; vomiting; restlessness, moaning and sighing; alternate paleness and flushing of the face; tongue white; bowels constipated.

"V. S. ℥vj. En. Purg. Pil. ex Cal. et Ex. Col. C.

"13th. Blood dense, and slightly buffed; headach relieved by the bleeding; three dejections, yellow and greenish; pulse 114; skin cooler; some rest; no appetite; moaning and sighing; shooting pains in the occiput and forehead.

"Hirud. xij. pone aures. Rep. Pil. et En. Purg.

"14th. Temporary ease from the application of the leeches; rest disturbed by the headach; frequent sighing and starting; faintishness; slight epistaxis this morning; pulse 110, strong; skin hot and dry; vomiting; throbbing of the temples; two dejections, yellowish; unine high-coloured.

"V. S. ℥vj. statim. Vespere Hirud. xv. temporibus. Mist. Cath. Habt. Baln. tepid. nocte maneque.

"15th. Some rest after the venesection; headach returned in the evening, and was relieved by the leeches; vomiting less frequent; skin softer and cooler; pulse 96; bowels free; urine turbid.

"Cal. gr. ij. 3tiis horis. Rep. Baln. tepid.

"16th. Return of headach this morning; better rest; pulse 100; slight perspiration after the bath; sighing and moaning less frequent; some return of appetite; fæces yellow and greenish.

"Hirud. xij. temporibus. Cr. Cal. M. Cath. et Baln. tepid.

"17th. Some alleviation of the symptoms.—Cr.

"19th. Gradual amendment.—Cr.

"22d. Ptyalism. Convalescent.—Omittr. Cal. M. Cath. p. r. n."

Upon this case, Dr. Mills remarks:—

"That the present was a case of Hydrocephalus in its first stage we may conclude from the following circumstances: the delicate and irritable frame, and great sensibility of the patient; the family predisposition; the pains of the head; the dilatation of the pupils, and intolerance of light; the weaknesses, sighing, moaning, and starting; the restlessness, vomiting and irregularity of the bowels; the absence of disease in every organ save the brain; the inutility of purgatives; the efficacy of general and local blood-letting; the cessation of a discharge from the ears which had previously subsisted: and, that the first stage of the complaint was inflammatory, we may conclude from the history of the symptoms and the good effects of depletion." 431.

"*Case* 2. In the month of September, 1813, I visited a fine boy, six years old, who complained of shooting pains in the forehead and occiput, of sickness, retching, and want of appetite; his temples throbbed; his face was alternately pale and flushed; the heat of the forehead, often bedewed with perspiration, was intense; the bowels were confined, and the urine was high-coloured; there was restlessness, sighing and moaning; the pupils were dilated, and very sensible to the application of light; the eyes were suffused, and the countenance was expressive of pain and distress; the pulse varied in strength and regularity, and in frequency from about 120 to 130.

"This state of excitement had existéd six or eight days, and was preceded by a fortnight's indisposition, which was ascribed to cold and indigestion. Six ounces of blood were immediately taken from the arm; three grains of calomel, mixed with eight of jalap, were given every fourth hour until they operated, and in the evening twelve leeches were applied to the temples. On the mornings of the three successive days the same quantity of blood was taken from the arm, and every evening the same number of leeches was applied to the temples; a cathartic was daily exhibited, and the tepid bath was employed morning and evening.

"By these means a considerable remission of the urgent symptoms was produced, and for four days the parents of the little patient supposed the danger to be past; the complaint, however, then recurred with violence, and it became necessary twice again to have recourse to general and local blood-letting, and to repeat the cathartic and the tepid bath. Three grains of calomel, combined with a small quantity of the watery extract of opium, were given every sixth hour until the gums became affected, and ipecacuanha was added in order to determine to the surface; the fæces varied in colour and consistence, being dark, hardened, greenish, yellow and slimy; and while the cathartic was operating considerable uneasiness was felt in the abdomen, augmented by pressure, and always mitigated by fomentations or the tepid bath. As this child was subject to eruptions of the head and a watery discharge from behind the ears, a drain was established in the vertex by means of lunar caustic, and kept open for nearly seven months. A fit regimen and country air were recommended, and these have prevented a return of the complaint."* 433.

Seeing that hydrocephalus often flows by an hereditary prerogative from father to son, and taints the constitutions of entire families; that it is so insiduous in its approach, and so rapid it in its progress; so perverse in its management, and so destructive in its issue; every caution should be observed that would tend, either to diminish the frequency of its occurrence, or lessen the violence of its action. The children of families predisposed to it should be preserved in the best state of general health; their intestinal secretions should be daily noticed; and, immediately that either languor, anorexia, vacancy of aspect, headach, or aversion to exercise betrays itself, every moment ought to be one of watchfulness, and such treatment should be instituted as will be best calculated to meet whatever event may occur. Much mischief may result in doubtful cases from delay, but little can arise from alertness; and, although the issue may occasionally prove the groundlessness of our fears, it is much better to be guilty of an error which may reflect upon our judgment, than of one which may destroy a valuable life.

"Our great objects are, to maintain the equilibrium of the circulation, and preserve the viscera in a healthy condition; this is to be done by supporting the actions of the cutaneous vessels, and preserving a healthy state of the secretions by the establishment of issues, the use of cold and tepid baths, exercise, warm clothing, proper diet and medicines, and living in a pure air." 458.

Issues and setons are especially useful when, from any suppressed eruption

"* Eight additional cases of recovery from supposed Hydrocephalus were submitted to the Association by Dr. Mills, which the Committee of Publication, for want of space, are reluctantly obliged to withhold." 433.

or discharge, the balance of the circulation is lost, and the head becomes encumbered with blood. A caustic issue formed on the vertex has been very highly recommended; but there is a serious inconvenience attached to all such remedies; when once established, we often run the greatest hazard in removing them. If they be not persevered in, until the constitution have so gained upon the predisposition to disease, that little fear remains of its approach; to dry them up, would in nine cases out of ten, either immediately produce what we had been endeavouring to prevent, or so increase the predisposition to it that our prophylaxis would be rendered much more troublesome.

After speaking highly of the cold bath as a preventive, he adds :—

"In delicate habits, where the cold bath is not admissible, recourse may be had to the tepid sea-water bath, which often produces all the good expected from the cold. This bath may be used every second or third day, and the patient may remain in it from fifteen to thirty minutes; meanwhile the body and extremities should be constantly rubbed with the flesh-brush; in situations remote from the sea, fresh-water, strongly impregnated with rock-salt, will answer instead of sea-water. In a variety of instances the shower-bath, cold or tepid, according to circumstances, will prove highly efficacious." 460.

The diet should be light and easily digested, and neither repletion nor abstinence allowed; the bowels should be carefully observed, as a very slight derangement in the cerebral functions will generally produce in them palpable disorder; the temperature of the body should be preserved moderate and equable; the head should be kept elevated during sleep, and no solid food taken immediately before it; nothing should be permitted as dress, which might obstruct any part of the circulation, especially that devoted to the head; the purest air should be inhaled; regulated exercise should be taken, and every passion and emotion of the mind should be kept within proper limits.

Our extracts have been hitherto selected for the sake of our readers; let our author participate in the object of the last. While the citation of the following passage is only justice to the one, it will enable the other to appreciate the value of patient research and successful labor, when contrasted with the chamber-spun fineries of library-makers, and the indigested fancies of Æsculapian poets :—

"When the reader examines the nature of the present undertaking, and is informed that every case was taken from my own private practice, and that I was present at every dissection, one excepted, he will readily appreciate the labour and anxiety attendant on such a pursuit, the difficulties I had to encounter, and the prejudices and feelings to combat and appease. In truth I should have shrunk from an inquiry, at once painful and laborious, had I not been impressed with a conviction that through this channel alone I could become acquainted with the seat and nature of the disease, and a just and rational mode of treatment.

"For the benefit of those who may be desirous to pursue the same path, with regard to Hydrocephalus, or any other complaint, I shall beg leave to state, in a few words, the plan I pursued. During my attendance on each patient I took notes daily of the case; if death followed I noted, assisted by the surgeon, the morbid appearances; on my return home, while the subject was fresh in my memory, I made a fair copy of the case and dissection, wrote the observations to which these gave rise, and the names of the surgeons by whom the bodies were examined." 467.

We have now done with Dr. Mills, and we feel refreshed after our review of his very valuable Essay. It presents no parade of learning, nor display of theory. Truth comes forward to you in her native and most becoming garb of unostentatious modesty, aware that the more she is examined, the more she will please; and that, although the *naiveté* of her exterior may repel the vain, the substantial *materiel* of her internal qualities, will constitute a sufficient attraction to the honest inquirer after knowledge. If Dr. Mills can, with justice to his health and circumstances, persevere in his *synthetic* labours, by every such contribution as the present, he will lay the profession under a fresh obligation, and insert a new leaf into his wreath of laurel.

